# Development of a HPLC-MS/MS Method to Determine 11 Bioactive Compounds in Tongmai Yangxin Pill and Application to a Pharmacokinetic Study in Rats

**DOI:** 10.1155/2018/6460393

**Published:** 2018-09-26

**Authors:** Jiayuan Shen, Juan Wei, Li Li, Huizi Ouyang, Yanxu Chang, Xiaopeng Chen, Jun He

**Affiliations:** Tianjin State Key Laboratory of Modern Chinese Medicine, Tianjin University of Traditional Chinese Medicine, Tianjin 300193, China

## Abstract

A sensitive and reliable HPLC-MS/MS method has been developed and validated for simultaneous determination of eleven bioactive compounds (rhein, emodin, stilbene glycoside, liquiritin, ononin, verbascoside, gallic acid, schisandrin, liquiritigenin, glycyrrhizic acid, and isoliquiritigenin) in rat plasma after oral administration of Tongmai Yangxin Pill. The collected plasma samples were prepared by liquid-liquid extraction with ethyl acetate after acidification. Eleven compounds were separated on a CORTECS™ C18 column with mobile phases consisting of 0.1% formic acid in deionized water and acetonitrile. The flow rate was 0.3 mL/min. The detection was performed on a tandem mass system with an electrospray ionization (ESI) source in both positive and negative ionization using multiple-reaction monitoring (MRM) mode. The calibration curves were linear over the range of 8-2000 ng/mL for glycyrrhizic acid; 4-1000 ng/mL for liquiritin; 0.8-200 ng/mL for emodin, gallic acid, ononin, schisandrin, and stilbene glycoside; 0.4-100 ng/mL for isoliquiritigenin, liquiritigenin, rhein, and verbascoside, respectively. The intra- and interday precision of the analytes were less than 9.3% and 8.5%. The intra- and interday accuracy were in the range of -14.0% to 10.3% and -6.5% to 9.6%. Meanwhile, the extraction recovery of the analytes in plasma samples ranged from 85.2% to 109.1% and matrix effect from 89.2% to 113.4%. The developed method was successfully applied to the pharmacokinetics of eleven bioactive compounds in rat plasma after oral administration of Tongmai Yangxin Pill prescription.

## 1. Introduction

Traditional Chinese Medicine (TCM) is a precious treasure of nature. TCMs have been used in clinical for thousands of years and attracting rising attentions due to the treatment of divers diseases successfully with minimum side effects [1–3]. TCM prescription, the most commonly used form in clinical medication, has multicomponents and multitargets [4]. The multicomponents and multitargets are superiority and characteristic of TCM pharmacological actions [5].

Tongmai Yangxin Pill (TMYX) is a traditional Chinese patent medicine documented in Chinese Pharmacopoeia. The prescription was developed from a well-known herb pair “GuiZhi-GanCao” documented in “Shang-Han-Lun” by Zhongjing Zhang in the Eastern Han Dynasty. The prescription consists of eleven herbs including* Radix rehmanniae, Caulis spatholobi, Radix glycyrrhizae, Ramulus cinnamomi, Radix ophiopogonis, Radix polygoni multiflori preparata, Asini corii colla, Fructus schisandrae, Radix codonopsis, Capapax et Plastrum testudinis, and Fructus jujubae *[6]. TMYX has been used to treat coronary heart disease, arrhythmia, chest pain, and angina for several decades [7–9]. Modern pharmacological studies show that TMYX has a significant effect on heart disease. Four active flavonoid compounds (glyasperin A, glycycoumarin, licorisoflavan A, and licoisoflavone A) from TMYX were found to have satisfactory biological activity and promote proliferation and angiogenesis of human umbilical vein endothelial cells in zebrafish. [10]. Meanwhile, Liu et al. reported that five fractions of TMYX were found to exert antiepithelial-mesenchymal transition activity [11]. Tao's results indicated that six active ingredients with high R values (gomisin D, schisandrin, glycyrrhizic acid, stilbene glycoside, formononetin, and ononin) exert antiinflammatory effects in a dose-dependent manner [12].

The pharmacological effects of TMYX are based on the diverse chemical composition. Chen reported 80 compounds were identified or surmised using HPLC-MS, including 23 flavones and their glucuronides, 6 phenethyl alcohol glycosides, 20 triterpene saponins, 15 lignans, and 18 other compounds [13]. Fan characterized 40 absorbed bioactive components after oral administration of TMYX in rat serum by UPLC/Q-TOF-MS [14]. The 40 components including 2 from* Radix rehmanniae*, 10 from* Radix codonopsis*, 2 from* Radix ophiopogonis*, 2 from* Ramulus cinnamomi*, 19 from* Radix glycyrrhizae*, 2 from* Radix polygoni multiflori preparata*, 5 from* Caulis spatholobi*, 1 from* Fructus jujubae,* and 1 from* Fructus schisandrae*, some of which are overlapped. Most of the ingredients exert anti-inflammatory and antioxidation effects, furthermore exerting the cardiovascular protective effect. The team also determines the concentrations of liquiritin, liquiritigenin, isoliquiritigenin, glycyrrhizic acid, and glycyrrhetinic acid in rat plasma following oral administration of* Radix glycyrrhizae* or the combination of* Radix glycyrrhizae* and* Ramulus cinnamomi *by HPLC-UV. However, there is no publication that reports the pharmacokinetic study of TMYX.

Pharmacokinetics (PK) of TCMs is a branch of the pharmacology of TCMs. PK of TCMs focuses on quantitatively studies the laws of drug absorption, distribution, metabolism, and excretion in a living organism. PK study of multicomponents of TCM prescription has been one of the important research aspects of modernization of TCMs [1]. The PK data could elucidate the substance basis and reveal the scientific connotation of TCMs. It also plays an important role in the creation of new Chinese medicines, the improvement of dosage forms, and the mechanism of formulation mechanism. In the present study, a reliable and sensitive HPLC-MS/MS method was first developed and applied to the pharmacokinetic study of 11 bioactive components including rhein, emodin, stilbene glycoside, liquiritin, ononin, verbascoside, gallic acid, schisandrin, liquiritigenin, glycyrrhizic acid and isoliquiritigenin in rats after oral administration of TMYX. The pharmacokinetic characteristics of the main chemical components of TMYX in rats were revealed, which would provide a theoretical basis for use of TMYX in clinical.

## 2. Experimental

### 2.1. Chemicals, Reagents, and Materials

Methanol (chromatographic purity) and acetonitrile (chromatographic purity) were purchased from Merck Co., Ltd. Formic acid (chromatographic purity) was obtained from ROE Co., Ltd. Ultra-pure water was prepared with a Milli-Q water purification system (Millipore, Milford, MA, USA). Rhein, emodin, stilbene glycoside, liquiritin, ononin, verbascoside, gallic acid, schisandrin, liquiritigenin, glycyrrhizic acid, isoliquiritigenin, and icariin were purchased from Chengdu Must Bio-Technology Co., Ltd. (Chengdu, China). TMYX were supplied by Tianjin Zhongxin Pharmaceutical Group Co., Ltd.

### 2.2. Chromatographic and Mass Spectrometry Conditions

The HPLC-MS/MS system consists of an Agilent 1200 high-performance liquid chromatography coupled with an Aglient 6430 series triple quadrupole mass spectrometer with an electrospray ionization (ESI) source. The chromatographic separation was achieved on a CORTECS C18 column (4.6 mm × 150 mm, 2.7 *μ*m), and the column temperature was kept at 30°C. Mobile phases which consisted of 0.1% formic acid in water (A) and acetonitrile (B) were used in the following gradient elution method: 0-10 min, 10%-85% B; 10-13 min, 85%-95% B; 13-19 min, 95%-95% B. The flow rate was set at 0.3 mL/min, and the injection volume was 10 *μ*L. All data were analyzed by Mass Hunter workstation software (Agilent Technologies, USA).

The mass spectrometer was carried out in both positive and negative ionization multiple-reaction monitoring (MRM) mode. The source parameters were as follows: the capillary voltage set at 300 V for positive ionization mode and -300 V for negative ionization mode, the drying gas temperature was 320°C, the flow was 11 L/min, and nebulizing gas pressure was 30 psi. The precursor and production of the ingredients and MRM parameters were displayed in Table 1.

### 2.3. TMYX Extract Preparation

**Figure 1:**
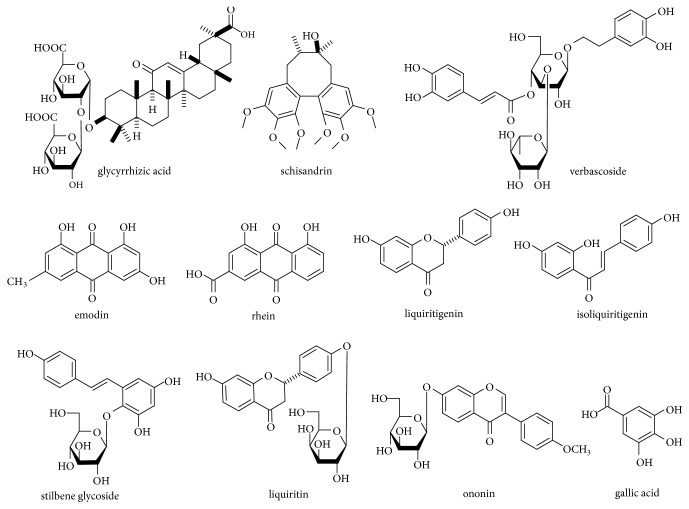
Chemical structures of eleven components.

TMYX extract was prepared as follows: A total of 100 g TMYX powder was accurately weighed and extracted twice under heat reflux by four times amounts of 60% ethanol (v/v) for 1 h per time. After that, the extraction solutions were filtered and mixed. The mixed solution was concentrated by evaporation under reduced pressure. And then the dried extracts was crushed to powder and kept in a desiccator until analysis. The extracts contains rhein, emodin, stilbene glycoside, liquiritin, ononin, verbascoside, gallic acid, schisandrin, liquiritigenin, glycyrrhizic acid, and isoliquiritigenin 0.4, 46.3, 231.6, 270.5, 161.1, 27.8, 71.8, 65.2, 16.9, 519.7, and 9.6 *μ*g/g, respectively. The structures of the compounds in the study were shown in Figure 1.

### 2.4. Calibration Solutions and Quality Control Samples Preparation

To make the stock solution, rhein, emodin, stilbene glycoside, liquiritin, ononin, verbascoside, gallic acid, schisandrin, liquiritigenin, glycyrrhizic acid, isoliquiritigenin, and icariin (internal standard solution) were weighed separately and diluted with methanol to a final concentration of 1 mg/mL. The mixed standard solution was obtained by mixed appropriate volume of eleven stock solutions and diluted with methanol.

The calibration solutions were prepared by spiking the 20 *μ*L of the mixture standard solution and 20 *μ*L of IS into 100 *μ*L blank rat plasma. The final concentrations of the series analytes were at the range of 8-2000 ng/mL for glycyrrhizic acid; 4-1000 ng/mL for liquiritin; 0.8-200 ng/mL for emodin, gallic acid, ononin, schisandrin, and stilbene glycoside; and 0.4-100 ng/mL for isoliquiritigenin, liquiritigenin, rhein, and verbascoside.

Quality control (QC) samples at three concentrations (low, medium, and high concentration) were made up of appropriate mixed standard solutions with blank blood sample as calibration solutions to meet the required concentrations. All the solutions were kept at 4°C.

### 2.5. Plasma Sample Preparation

The plasma (100 *μ*L) was spiked with 20 *μ*L of methanol, 20 *μ*L of the IS (icariin, 1 *μ*g/mL), and 20 *μ*L of formic acid and then vortex-mixed. The mixture was extracted with 800 *μ*L of ethyl acetate by vortex mixing for 5 min at room temperature. After centrifugation at 14,000 rpm for 10 min, the supernatant was collected to a clean tube and evaporated to dryness under a nitrogen stream. The residue was reconstituted in 50 *μ*L of 50% methanol, vortexed for 5 min, and centrifuged at 14,000 rpm for 10 min. Finally, 10 *μ*L supernatant was injected into the LC-MS/MS system for analysis.

### 2.6. Method Validation

#### 2.6.1. Specificity

The specificity was assessed by analyzing blank blood samples from six different rats. Each plasma sample was evaluated for endogenous interference using the suggested extraction program and LC-MS/MS conditions above.

#### 2.6.2. Linearity and LLOQ

The linearity was achieved by assaying blank rat plasma with a serious of the mixed standard solution and IS in duplicate over 3 consecutive days. The calibration curves were plotted by the peak-area ratios (y) of analyte against internal standard versus the nominal concentration (x). The weight factor is 1/*x*. The lower limit of quantification (LLOQ) was evaluated according to the base line noise, defining a signal-to-noise ratio of about 10.

#### 2.6.3. Precision and Accuracy

The precision and accuracy were assessed by determining QC samples at low, medium, and high concentration levels (20, 200, and 2000 ng/mL for glycyrrhizic acid; 10, 100, and 1000 ng/mL for liquiritin; 2, 20, and 200 ng/mL for emodin, gallic acid, ononin, schisandrin, and stilbene glycoside; 1, 10, and 100 ng/mL for isoliquiritigenin, liquiritigenin, rhein, and verbascoside). All concentration levels were measured in six replicates. The precision and accuracy were tested once a day and repeated for 3 consecutive days with the standard calibration curve. Intra- and interday precision were defined as the relative standard deviation (RSD), while the accuracy was determined by the relative error (RE %).

#### 2.6.4. Extraction Recovery and Matrix Effect

The extraction recovery of eleven analytes at three concentration levels and IS were assayed by comparing the peak areas obtained from extracted samples with those of the postextracted samples. The matrix effect of the samples and IS were estimated by the peak-area ratios of the analytes in postextracted spiked samples to those obtained from unextracted samples. Both the extraction recovery and matrix effect were tested in six parallels.

#### 2.6.5. Stability

The stability of analytes in plasma samples was determined by analyzing QC samples of three concentration levels at different conditions: stored at autosampler for 12 h, at room temperature for 6 h, under three freeze-thaw cycles and stored at -70°C for 14 days. All stability studies were measured in six replicates.

### 2.7. Pharmacokinetic Study

Male Sprague–Dawley rats (230-250 g) were obtained from Beijing HFK Experimental Animal Technology Co., Ltd. The rats were maintained under the control environmental conditions and they fasted for 12 h with free access to water before the experiments. TMYX extracts were dissolved in CMC-Na and administrated orally to rats at 8.3 g/kg. The blood samples (200 *μ*L) were collected from the rat fossa orbitalis vein at 0, 0.03, 0.083, 0.17, 0.25, 0.5, 1, 2, 4, 6, 8, 10, 12, 24, 36, and 48 h after oral administration to heparinized tubes. And then the blood samples were centrifuged at 7,000 rpm for 10 min to get the plasma sample immediately. Finally, the plasma obtained was stored at −70°C until analysis. Pharmacokinetic parameters were calculated by the computer program “Drug and Statistics 2.0” (DAS 2.0) (Medical College of Wannan, China).

## 3. Result and Discussion

### 3.1. HPLC-MS/MS Method

Several mobile phases were tested. Comparing with a gradient mobile phase system with acetonitrile-water or methanol-water, the mobile phase contains 0.1% formic acid in water could improve the peak shape and increase the signal response of the analytes. Thus, we chose the water containing 0.1% formic acid as the mobile phase.

The standard solutions of analytes and internal standard were injected into the mass spectrometer, respectively. The ononin and schisandrin were tested in positive ion mode, while others in negative. The optimized precursor-to-production transitions were monitored at 238.9→211.0 for rhein, 269.0→225.0 for emodin, 405.2→242.8 for stilbene glycoside, 417.1→255.0 for liquiritin, 431.2→269.1 for ononin, 623.0→161.1 for verbascoside, 169.0→125.1 for gallic acid, 433.3→384.2 for schisandrin, 255.3→119.1 for liquiritigenin, 821.1→350.5 for glycyrrhizic acid, 255.1→118.9 for isoliquiritigenin, and 721.0→513.2 for IS. All data were shown in Table 1.

### 3.2. Sample Preparation

In the experiment, we tested two methods to dispose the plasma sample including liquid-liquid extraction (LLE) and protein precipitation (PPT). The results show that both the recovery and matrix effects of the methods meet the requirements for the determination of biological samples and the endogenous substances do not interfere with the analysis. However, the method of PPT showed lower extraction efficiency and higher matrix effect relatively. Consequently, we selected liquid-liquid extraction (LLE) with ethyl acetate to prepare the sample.

### 3.3. Method Validation

#### 3.3.1. Specificity

**Figure 2:**
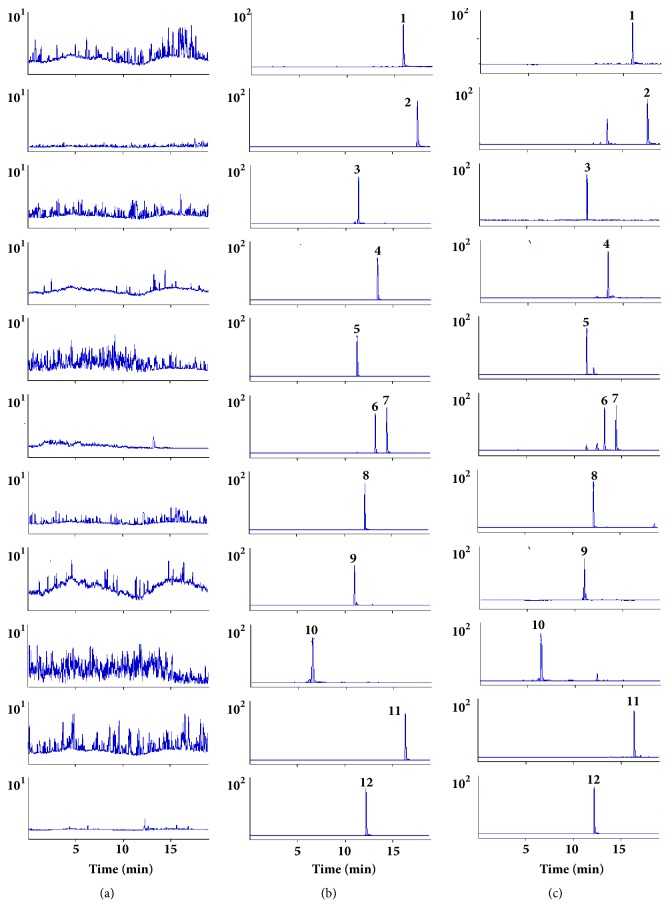
MRM chromatograms of eleven analytes. rhein** (1)**, emodin** (2)**, stilbene glycoside** (3)**, glycyrrhizic acid** (4)**, liquiritin** (5)**, liquiritigenin** (6)**, isoliquiritigenin** (7)**, ononin** (8)**, verbascoside** (9)**, gallic acid** (10)**, schisandrin** (11),** and IS** (12)**. (a) Blank plasma; (b) blank plasma spiked with the analytes and IS; (c) plasma sample after oral administration of TMYX extract.

The specificity was estimated by comparing chromatograms of blank blood samples from six different rats with blood samples containing analytes. The representative chromatograms of blank blood sample, blank blood sample containing eleven analytes and IS, and plasma sample obtained from a rat after oral administration of TMYX extracts were accessed. The retention time of rhein, emodin, stilbene glycoside, liquiritin, ononin, verbascoside, gallic acid, schisandrin, liquiritigenin, glycyrrhizic acid, isoliquiritigenin, and IS were 15.93, 17.63, 11.34, 11.31, 12.21, 11.01, 6.48, 16.41, 13.21, 13.34, 14.41, and 12.19 min, respectively. Based on the chromatograms, endogenous substances in plasma samples do not interfere with the determination of analytes and IS. The chromatograms were shown in Figure 2.

#### 3.3.2. Linearity and Sensitivity

The results of calibration curves, linear ranges, correlation coefficients, and LLOQs were displayed in Table 2. The plasma calibration curves were constructed within the range of 8-2000 ng/mL for glycyrrhizic acid; 4-1000 ng/mL for liquiritin; 0.8-200 ng/mL for emodin, gallic acid, ononin, schisandrin, and stilbene glycoside; 0.4-100 ng/mL for isoliquiritigenin, liquiritigenin, rhein, and verbascoside.

The LLOQs for 11 analytes in plasma sample were less than 8 ng/mL, which are sensitive enough for the pharmacokinetic studies.

#### 3.3.3. Precision and Accuracy

In this assay, the intra- and interday precision and accuracy were analyzed at three concentration levels in six replicates. The data were displayed in Table 3. The RSD of intra- and interday precision were between 0.7 and 9.3%. The RE of accuracy was within ±14.0%. The results suggest that the method is accurate and repeatable for analysis of all analytes in rat plasma.

#### 3.3.4. Extraction Recovery and Matrix Effect

All data of the extraction recovery and matrix effect were summarized in Table 4. The extraction recovery of 11 ingredients at three concentration levels were in the scope of 85.2-109.1%. The matrix effects of all analytes ranged from 89.2 to 113.4%. The data which manifests the procedure of the experiment is efficient and the matrix effects could be ignored.

#### 3.3.5. Stability

To investigate the stability of analytes, QC samples of three concentrations levels under the different storage conditions were tested, including stored at autosampler for 12 h after preparation, at room temperature for 6 h, at three freeze-thaw cycles, and at -70°C for 14 days. As shown in Table 5, the results suggest that the analytes are stable in the above conditions.

### 3.4. Pharmacokinetic Study

**Figure 3:**
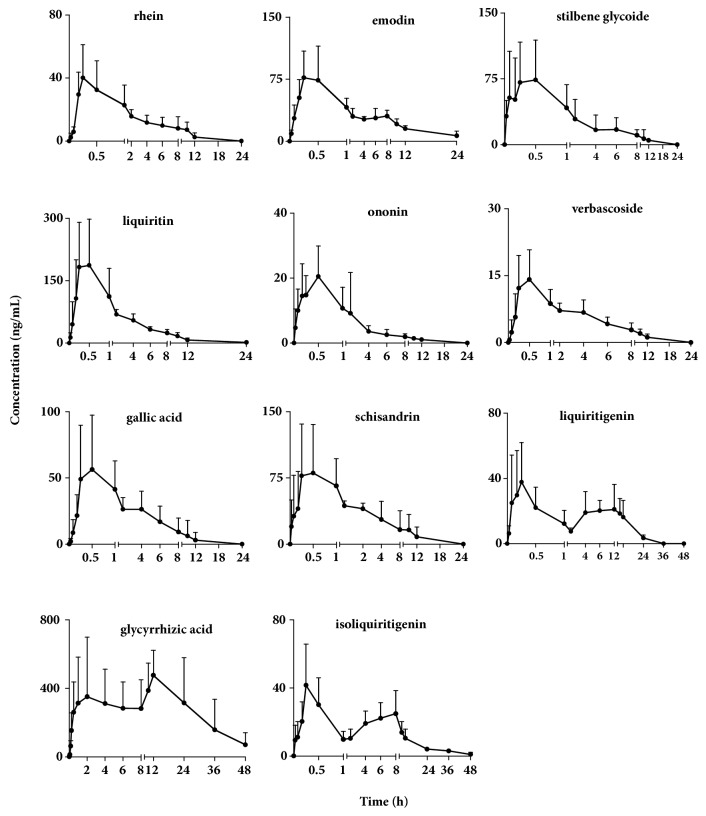
Mean plasma concentration-time curves of rhein, emodin, stilbene glycoside, liquiritin, ononin, verbascoside, gallic acid, schisandrin, liquiritigenin, glycyrrhizic acid, and isoliquiritigenin after oral administration of TMYX extract (mean ± SD, n=6).

The validated LC-MS/MS method was applied to the pharmacokinetic study of the eleven analytes in rat blood sample after oral administration of TMYX at a single dose of 8.3 g/kg. The major pharmacokinetic parameters were demonstrated in Table 6. And the mean plasma concentration-time profiles of the eleven active ingredients were shown in Figure 3.

The* T*_*max*_ of rhein, emodin, stilbene glycoside, liquiritin, ononin, verbascoside, gallic acid, and schisandrin are 0.36±0.31 h, 0.32±0.14 h, 0.50±0.31 h, 0.50±0.46 h, 0.46±0.10 h, 0.37±0.18 h, 0.75±0.67 h, and 1.00±0.92 h, respectively. The* T*_*max*_ shows that eight ingredients are absorbed rapidly. The double peaks are detected in the mean plasma concentration-time profiles for liquiritigenin, glycyrrhizic acid, and isoliquiritigenin. The first peak of three ingredients appeared at 0.20 h, 2.13 h, and 0.28 h and the second peak at 7.20 h, 17.67 h, and 7.00 h, respectively. It might result from hepatoenteral circulation. One peak plasma concentration-time profiles of liquiritigenin, isoliquiritigenin, and glycyrrhizic acid were reported in rat plasma following oral administration of* Radix glycyrrhizae* or the combination of* Radix glycyrrhizae* and* Ramulus cinnamomi* [8]. The differences may come from the effects of other herbs in TMYX which require further experiment. Besides double peaks, the half-life of elimination (*t*_*1 /2*_) of the rhein, emodin, gallic acid, glycyrrhizin, stilbene glycoside, verbascoside, formononetin, and schisandrin range from 1.53 h to 4.21 h, which suggests that these eight analytes in rat blood sample are eliminated fleetly after oral administration. The similar pharmacokinetic trend of schisandrin was reported after oral administration of* Fructus schisandrae* aqueous extract [15]. It was also reported that one peak was observed for emodin and gallic acid in rat dosed processed* Radix polygoni multiflori *[16]. The* t*_*1 /2*_ of the liquiritigenin, glycyrrhizic acid, and isoliquiritigenin are 12.70 h, 18.19 h, and 13.46 h, which indicates that the ingredient has a longer treatment time, especially isoliquiritigenin and glycyrrhizic acid, which can still be detected* in vivo* at 48 h.

## 4. Conclusion

In our experiment, we developed a method of HPLC-MS/MS for detecting the rhein, emodin, stilbene glycoside, liquiritin, ononin, verbascoside, gallic acid, schisandrin, liquiritigenin, glycyrrhizic acid, and isoliquiritigenin in rat plasma. The pharmacokinetic parameters would be helpful for the further development and usage in clinical of TMYX. The 11 active ingredients of TMYX, which were absorbed into plasma, would provide data support for quality control improvement.

## Figures and Tables

**Table 1:** Mass spectra properties of 11 analytes and IS.

Compounds	Precursor Ion (*m/z*)	Product Ion (*m/z*)	Frag. (V)	C.E. (V)
rhein	238.9	211.0	140	-10
emodin	269.0	225.0	145	-20
stilbene glycoside	405.2	242.8	145	-13
liquiritin	417.1	255.0	145	-13
ononin	431.2	269.1	10	13
verbascoside	623.0	161.1	116	-33
gallic acid	169.0	125.1	123	-12
schisandrin	433.3	384.2	100	14
liquiritigenin	255.3	119.1	121	-24
glycyrrhizic acid	821.1	350.5	125	-40
isoliquiritigenin	255.1	118.9	106	-23
icariin (IS)	721.0	513.2	145	-10

**Table 2:** Calibration curves, correlation coefficients, linear ranges, and LLOQ of the analytes.

Compounds	Calibration curves	Correlation coefficients (r)	Linear range (ng/mL)	LLOQ (ng/mL)
rhein	y = 0.0954 x + 1.5911	0.9966	0.4 – 100	0.4
emodin	y = 1.2189 x + 0.0135	0.9906	0.8 – 200	0.8
stilbene glycoside	y = 6.6409 x + 0.0293	0.9991	0.8 – 200	0.8
gallic acid	y = 0.9987 x + 0.0104	0.9987	0.8 – 200	0.8
ononin	y = 5.4632 x + 0.0058	0.9995	0.8 – 200	0.8
verbascoside	y = 0.3514 x + 1.9984	0.9965	0.4 – 100	0.4
liquiritin	y = 1.1970 x + 0.0085	0.9965	4 – 1000	4
schisandrin	y = 2.5769 x + 0.0020	0.9980	0.8 – 200	0.8
liquiritigenin	y = 2.2148 x + 0.0140	0.9997	0.4 – 100	0.4
glycyrrhizic acid	y = 0.0917 x - 0.0028	0.9982	8 – 2000	8
isoliquiritigenin	y = 3.1496 x + 0.0064	0.9986	0.4 – 100	0.4

**Table 3:** Precision and accuracy of 11 analytes in rat plasma (n=6).

compounds	Spiked Concentration (ng/mL)	Intra-day			Inter-day		
		Measured concentration (ng/mL)	Accuracy (RE, %)	Precision (RSD, %)	Measured concentration (ng/mL)	Accuracy (RE, %)	Precision (RSD, %)
rhein	1	1.00±0.01	0.5	1.2	0.99±0.01	-1.0	1.0
	10	10.16±0.52	1.6	5.1	10.04±0.34	0.5	3.4
	100	106.60±1.52	6.6	1.4	101.83±4.74	1.8	4.7
emodin	2	2.01±0.10	0.3	4.9	2.00±0.05	0.1	2.7
	20	21.72±0.57	8.6	2.6	20.47±1.23	2.3	6.0
	200	208.21±1.71	4.1	0.8	201.43±8.99	0.7	4.5
stilbene glycoside	2	1.99±0.06	-0.4	3.0	2.05±0.03	2.3	1.6
	20	20.17±0.76	0.8	3.8	19.91±0.73	-0.5	3.7
	200	192.33±2.26	-3.8	1.2	192.80±6.57	-3.6	3.4
gallic acid	2	1.96±0.05	-2.0	2.6	2.06±0.05	3.1	2.4
	20	20.00±0.31	0.1	1.6	20.27±0.61	1.4	3.0
	200	186.32±1.66	-6.8	0.9	188.68±3.27	-5.7	1.7
ononin	2	1.96±0.04	-1.9	2.0	1.95±0.03	-2.6	1.4
	20	20.59±0.88	3.0	4.3	19.62±1.09	-1.9	5.6
	200	208.21±1.71	4.1	0.8	202.11±5.92	1.1	2.9
verbascoside	1	0.92±0.01	-7.8	1.5	0.96±0.03	-3.9	3.6
	10	9.82±0.36	-1.8	3.7	9.66±0.13	-3.4	1.4
	100	102.66±2.89	2.7	2.8	102.03±3.01	2.0	3.0
liquiritin	10	9.45±0.08	-5.5	0.8	9.89±0.49	-1.1	4.9
	100	110.26±3.44	10.3	3.1	109.58±2.85	9.6	2.6
	1000	1015.89±23.42	1.6	2.3	990.69±29.65	-0.9	3.0
schisandrin	2	2.00±0.04	0.2	2.0	1.89±0.06	-5.4	3.4
	20	21.49±0.50	7.5	2.3	20.63±0.70	3.2	3.4
	200	199.93±2.23	-0.1	1.1	197.48±3.11	-1.3	1.6
liquiritigenin	1	0.86±0.01	-14.0	1.4	0.93±0.08	-6.5	8.5
	10	10.37±0.96	3.7	9.3	9.41±0.50	-5.9	5.3
	100	106.60±1.52	6.6	1.4	98.95±7.06	-1.1	7.1
glycyrrhizic acid	20	18.72±0.53	-0.1	2.8	19.21±0.49	-4.0	2.6
	200	218.30±1.48	0.1	0.7	199.59±15.20	-0.2	7.6
	2000	2123.83±59.62	0.1	2.8	1980.71±75.51	-1.0	3.8
isoliquiritigenin	1	0.98±0.02	-2.1	2.1	0.98±0.01	-1.6	1.5
	10	9.82±0.14	-1.8	1.5	9.72±0.18	-2.8	1.8
	100	99.74±0.87	-0.3	0.9	100.71±3.08	0.7	3.1

**Table 4:** Extraction recoveries and matrix effects of the analytes (n=6).

Compounds	Spiked concentration (ng/mL)	Extraction recovery (%)	RSD (%)	Matrix effect (%)	RSD (%)
rhein	1	96.2±7.4	7.7	104.1±5.6	5.4
	10	99.3±3.9	3.9	100.1±2.7	2.7
	100	101.4±5.2	5.1	92.6±3.7	4.0
emodin	2	98.8±3.4	3.5	107.7±2.6	2.4
	20	96.2±2.5	2.6	103.6±0.8	0.8
	200	86.1±4.2	4.9	104.8±2.9	2.8
stilbene glycoside	2	98.9±1.9	1.9	100.7±2.2	2.2
	20	99.3±4.0	4.0	94.6±2.2	2.3
	200	96.7±5.6	5.8	98.7±0.8	0.9
gallic acid	2	99.1±3.1	3.2	110.4±5.8	5.2
	20	100.9±2.5	2.5	98.3±2.0	2.0
	200	85.2±3.0	3.5	108.1±1.3	1.2
ononin	2	91.8±2.7	2.9	98.6±2.8	2.8
	20	89.0±3.8	4.3	89.9±0.7	0.8
	200	93.1±5.2	5.6	91.7±0.9	1.0
verbascoside	1	93.1±4.0	4.3	103.6±3.3	3.1
	10	97.0±4.7	4.8	100.0±2.1	2.1
	100	92.5±6.1	6.6	98.9±2.3	2.4
liquiritin	10	105.7±4.5	4.3	98.8±1.2	1.2
	100	109.1±4.0	3.6	100.1±6.4	6.4
	1000	99.5±7.6	7.7	95.2±5.5	5.8
schisandrin	2	98.8±4.1	4.2	96.8±2.6	2.7
	20	106.3±1.1	1.0	93.0±1.7	1.8
	200	101.0±6.0	5.9	90.4±0.7	0.8
liquiritigenin	1	99.7±3.5	3.6	113.4±2.8	2.5
	10	99.5±2.7	2.7	89.2±1.3	1.5
	100	96.3±3.4	3.5	99.8±1.8	1.8
glycyrrhizic acid	20	100.9±7.5	7.4	101.6±6.8	6.7
	200	97.5±2.1	2.1	102.5±1.3	1.3
	2000	87.6±1.6	1.8	103.7±2.1	2.0
isoliquiritigenin	1	98.0±5.5	5.6	105.73±2.2	2.1
	10	96.5±2.0	2.1	98.0±3.6	3.6
	100	85.9±5.7	6.7	97.3±3.1	3.2

**Table 5:** Stability of all analytes in rat plasma (n=6).

Compounds	Spiked Concentration (ng/mL)	room temperature for 6 h		three freeze-thaw cycles		auto-sampler for 12 h		-70°C for 14 days	
		Measured Concentration (ng mL^−1^)	RSD (%)	Measured Concentration (ng mL^−1^)	RSD (%)	Measured Concentration (ng mL^−1^)	RSD (%)	Measured Concentration (ng mL^−1^)	RSD (%)
rhein	1	0.97±0.02	2.0	0.98±0.03	2.8	0.97±0.01	0.8	0.95±0.05	5.4
	10	10.23±0.04	0.4	8.89±0.08	0.9	9.86±0.09	0.9	8.71±0.11	1.3
	100	101.97±1.07	1.1	86.7±1.33	1.5	97.32±1.05	1.1	85.95±0.39	0.5
emodin	2	1.95±0.05	2.3	2.00±0.02	0.9	1.93±0.02	0.8	1.86±0.03	1.6
	20	21.14±0.99	4.7	19.21±0.33	1.7	19.88±1.81	9.1	18.79±0.18	0.9
	200	207.49±1.27	0.6	197.74±5.65	2.9	200.50±11.66	5.8	185.45±0.98	0.5
stilbene glycoside	2	1.82±0.01	0.8	2.00±0.03	1.6	1.89±0.00	0.1	1.99±0.01	0.3
	20	20.88±1.19	5.7	19.87±1.43	7.2	19.00±0.64	3.4	20.01±0.31	1.6
	200	202.79±12.80	6.3	200.45±2.10	1.1	182.40±1.23	0.7	203.34±1.39	0.7
gallic acid	2	1.97±0.03	1.5	1.93±0.04	1.8	2.03±0.05	2.3	1.99±0.04	2.0
	20	20.77±1.09	5.3	20.08±0.51	2.5	19.27±0.35	1.8	19.26±0.22	1.2
	200	205.97±2.14	1.0	196.77±1.09	0.6	184.54±2.46	1.3	199.19±1.80	0.9
ononin	2	2.02±0.03	1.4	1.85±0.01	0.7	1.83±0.01	0.4	1.95±0.01	0.5
	20	20.57±0.26	1.2	18.71±0.26	1.4	18.84±0.62	3.3	18.51±0.15	0.8
	200	216.32±0.91	0.4	191.28±2.36	1.2	195.34±4.30	2.2	200.23±3.89	1.9
verbascoside	1	0.92±0.01	1.0	1.09±0.01	0.9	1.05±0.02	1.8	1.01±0.02	2.3
	10	9.57±0.21	2.2	10.17±0.90	8.9	9.50±0.04	0.5	9.81±0.22	2.2
	100	103.95±1.33	1.3	108.42±1.17	1.1	93.52±0.27	0.3	107.40±1.47	1.4
liquiritin	10	9.85±0.09	1.0	9.26±0.16	1.7	10.26±0.11	1.1	10.04±0.12	1.2
	100	108.97±1.51	1.4	109.67±1.86	1.7	108.02±0.66	0.6	105.05±1.73	1.7
	1000	1076.07±10.27	1.0	952.89±11.22	1.2	869.12±14.52	1.7	1021.69±19.98	2.0
schisandrin	2	2.00±0.04	1.8	1.71±0.01	0.6	1.83±0.02	1.1	1.77±0.03	1.7
	20	21.15±0.50	2.4	18.28±0.23	1.2	18.35±0.09	0.5	17.86±0.20	1.1
	200	194.61±1.13	0.6	189.32±1.74	0.9	196.02±2.10	1.1	183.83±1.00	0.6
liquiritigenin	1	0.87±0.01	0.9	0.89±0.02	1.8	0.87±0.01	1.1	0.88±0.02	2.1
	10	10.82±0.58	5.3	8.99±0.07	0.8	8.63±0.23	2.7	9.26±0.08	0.9
	100	112.69±1.30	1.2	92.59±0.63	0.7	92.65±0.68	0.7	91.88±0.86	0.9
glycyrrhizic acid	20	20.49±0.79	3.9	19.30±0.20	1.0	19.25±0.34	1.8	18.77±0.09	0.5
	200	203.22±3.32	1.6	188.63±3.57	1.9	200.96±3.93	2.0	200.33±2.98	1.5
	2000	2099.10±8.92	0.4	1869.00±42.55	2.3	1884.66±9.26	0.5	1834.54±29.34	1.6
isoliquiritigenin	1	0.99±0.01	1.5	0.89±0.02	2.4	1.00±0.02	1.7	0.89±0.01	1.4
	10	10.86±0.60	5.5	8.69±0.14	1.6	9.60±0.04	0.4	8.62±0.04	0.4
	100	106.11±0.78	0.7	100.58±0.91	0.9	97.47±1.99	2.0	100.74±2.95	2.9

**Table 6:** Pharmacokinetic parameters of 11 analytes after oral administration of TMYX extract (n=6).

Compounds	*T*_*max1*_ (h)	*T*_*max2*_ (h)	*C*_*max1*_ (ng/mL)	*C*_*max2*_ (ng/mL)	*t*_*1/2*_ (h)	*K*_*e*_ (1/h)	*AUC*_(0-*tn*)_ (h·ng/mL)	*AUC*_(0-*∞*)_ (h·ng/mL)
rhein	0.36±0.31		46.3±15.6		2.84±2.13	0.56±0.50	145.0±52.6	152.1±61.3
emodin	0.32±0.14		88.0±37.5		4.21±2.63	0.39±0.28	475.5±121.0	561.3±192.3
stilbene glycoside	0.50±0.31		99.1±27.1		1.99±1.31	0.45±0.24	283.8±189.1	297.5±185.3
liquiritin	0.50±0.46		199.9±120.2		2.16±1.44	0.77±0.54	637.2±220.2	651.1±219.5
ononin	0.46±0.10		14.4±6.8		2.85±1.40	0.37±0.25	58.2±20.2	69.4±28.5
verbascoside	0.37±0.18		23.4±8.7		1.53±0.67	0.56±0.31	51.4±29.8	61.0±29.8
gallic acid	0.75±0.67		56.9±42.8		3.01±1.35	0.27±0.12	214.7±136.7	238.4±172.8
schisandrin	1.00±0.92		92.6±53.0		2.73±1.54	0.34±0.19	410.8±238.9	473.4±321.2
liquiritigenin	0.20±0.07	7.20±3.35	37.8±24.1	21.0±15.3	12.70±7.04	0.50±0.34	314.8±129.8	332.6±142.1
glycyrrhizic acid	2.13±1.44	17.67±10.31	351.7±347.4	476.1±146.0	18.19±9.61	0.05±0.03	12743.5±5058.2	17327.3±10967.3
isoliquiritigenin	0.28±0.11	7.00±2.45	41.6±24.2	24.9±13.5	13.46±8.33	0.07±0.04	410.0±130.7	436.2±154.1

## Data Availability

The data used to support the findings of this study are available from the corresponding author upon request.
